# Induction of Labour in a District Hospital of Rural Nepal: A Descriptive Cross-sectional Study

**DOI:** 10.31729/jnma.8491

**Published:** 2024-03-31

**Authors:** Raju Chapagain, Roshan Basnet, Sandesh Rai, Diwakar Chaudhary, Isha Thapa

**Affiliations:** 1 District Hospital Tehrathum, Myanglung, Tehrathum, Nepal; 2 Nobel Medical College and Teaching Hospital, Biratnagar, Morang, Nepal

**Keywords:** *apgar score*, *induced*, *labour*, *misoprostol*

## Abstract

**Introduction:**

Induction of labour, a medical intervention before spontaneous onset, is employed when the risk of continuing pregnancy is elevated.Common indications include intrauterine growth restriction, preeclampsia, gestational diabetes, placental abnormalities, prelabor rupture of membranes, post-term pregnancy, and intrauterine foetal demise. The objective of this study was to find out the prevalence of induction of labour in a rural setting in Nepal.

**Methods:**

We conducted a descriptive cross-sectional study in the District Hospital Tehrathum using patients' record files from 14 January 2021 to 14 January 2023. Ethical approval was obtained from Nepal Health Research Council. Demographic variables were collected along with maternal outcomes which include indication of induction of labour, mode of delivery, indication of lower segment caesarean section and foetal outcomes include APGAR score at one and five minutes, birthweight and liquor colour. A total population sampling method was used in the study and 95% confidence Interval was used to calculate the point estimate.

**Results:**

Among 640 deliveries during the study period 118 (18.43%) (15.43-21.43, 95% Confidence Interval) underwent induction of labour. Sixty-three (53.4%) of the 118 patients who underwent induction of labour were primigravida.

**Conclusions:**

The prevalence of induction of labour was comparable with previous studies. Neonatal outcome, rate of vaginal and lower segment C-section deliveries after induction of labour using misoprostol is comparable with other studies.

## INTRODUCTION

Induction of labour refers to interventions, before spontaneous onset of labour aiming for vaginal delivery when the risk of continuing pregnancy is high.^1^ Common indications include foetal intrauterine growth restriction, preeclampsia, gestational diabetes mellitus, abruptio placentae, prelabor rupture of membranes, post-term pregnancy, and intrauterine foetal demise.^2^ Pharmacological methods can be misoprostol, dinoprostone and oxytocin.^3^

The rate of induction varies in various parts of the country from 10.5% to 19.7%.^4,5^ This study would be important to find the indication and outcome of induction of labour in rural district hospitals where General Practitioners and Advanced Skill Birth Attendants provide obstetric care. The result of our study would be of great help to guide the general practitioners and medical officers of our hospital and similar setting for induction of labour.

Our study aimed to assess the prevalence of labour induction in 15-bed district hospitals within a rural setting, where obstetric care is supervised by General Practice and Emergency Medicine physicians.

## METHODS

A descriptive cross-sectional study was conducted in District Hospital Tehrathum using secondary data sources. Patients' record files were reviewed from 14 January 2021 to 14 January 2023 and data were collected from 30 July 2023 to 15 August 2023. Ethical approval was obtained from the Nepal Health Research Council (NHRC) (Reference number: 110) and written consent was obtained from hospital administration for collection of data from the patient records.

The patient records with complete maternal data, neonatal data and singleton pregnancy were included in the study. However, patient records with incomplete data, multiple pregnancy, malpresentation, previous caesarean section delivery, out of hospital deliveries and referred out cases despite undergoing induction were excluded from the study. The sample size was calculated using the following formula:


$$\begin{array}{ll} \text{n} & ={\text{Z}}^{2}\times \frac{\text{p}\times \text{q}}{{\text{e}}^{\text{2}}}\\ & =1.96^{2}\times \frac{0.105\times 0.895}{0.05^{2}}\\ & =144 \end{array}$$

Where,

n = minimum required sample size
Z = 1.96 at 95% Confidence Interval (CI)
p = prevalence taken as 10.5%^4^
q = 1-p
e = margin of error, 5%

Minimum required sample size was 144.

Total population sampling method was used and all the deliveries during the study period were taken which were 640 in number. Routes of misoprostol used for induction of labour and maternal and foetal outcomes recorded in the files were taken. Maternal outcomes include mode of delivery, indications of those deliveries and incidence of induction for various indications and foetal outcomes include one and five minutes APGAR score, birth weight of baby and meconium stained liquor. The completeness of data was checked and then analysed in Microsoft Excel 2019. Descriptive statistics such as frequency and percentages were used to describe variables. Point estimate at 95% Confidence Interval was calculated.

## RESULTS

Among 640 pregnant women, 118 (18.43%) (15.4321.43, 95% Confidence Interval) is the prevalence of induction of labour during the study period. A total of, 38 (32.20%) patients belonged to the age group 18-22 years (Table 1).

**Table 1:** Demographic distribution of the patients (n = 118).

Age group (years)	n (%)
Less than 18	7 (5.93)
18 to 22	38 (32.20)
23 to 27	37 (31.36)
28 to 32	28 (23.73)
More than 32	8 (6.78)

Among 118 patients who underwent induction of labour, 63 (53.39%) were primigravida and 55 (46.61%) were multigravida. A total of 113 (95.76%) cases of the induction were done on pregnant women with gestational age more than 40 weeks. The indications for induction of labour seen were post-dated pregnancy in 109 (92.37%) followed by intrauterine foetal demise (IUFD) in 5 (4.24%) (Table 2).

**Table 2:** Indication of induction of labour (n = 118).

Indications	n (%)
Post-dated pregnancy	109 (92.37)
IUFD	5 (4.24)
Premature rupture of membrane	4 (3.39)

Among 118 patients, 94 (79.66%) were delivered via vaginal delivery, 24 (20.34%) delivered via lower segment Caesarean section (LSCS). The indications for LSCS among the patients who underwent induction of labour were failed induction in 16 (66.67%), followed by foetal distress in 5 (20.83%) and non-progress of labour in 3 (12.50%) patients (Table 3).

**Table 3:** Mode of delivery among gravidity of the patients (n = 118).

Gravidity	Mode of delivery	
	Vaginal delivery n(%)	Caesarean section n(%)
Primigravida (n = 63)	45 (71.43)	18 (28.57)
Multigravida (n = 55)	49 (89.09)	6 (10.90)

For induction of labour, tablet misoprostol was given 50 mcg by per oral route or 25 mcg by sublingual route 6 hours apart (Table 4).

**Table 4:** Mode of delivery and routes of misoprostol administered (n = 118).

Route of misoprostol	Mode of delivery	
	Vaginal delivery n(%)	Caesarean section n(%)
Per oral (n = 67)	48 (71.64)	19 (28.35)
Sublingual (n = 51)	46 (90.19)	5 (9.80)

Among the 94 vaginal deliveries done using induction of labour, 19 (20.21%), 34 (36.17%), 20 (21.28%), 11 (11.70%) and 10 (10.64%) occurred after 1, 2, 3, 4 and 5 doses of misoprostol respectively. A total of 13 (54.17%) of the LSCS was done after 4 doses of misoprostol. Among the 118 induced women, 101 (85.59%) had clear liquor and 17 (14.40%) had meconium-stained liquor. Majority of delivered babies had a birth weight between 2500 grams to 4000 grams which was 106 (89.83%). A total of 5 (4.24%) and 7 (5.93%) of the delivered babies had birth weights less than 2500 grams and more than 4000 grams respectively. All of the live births 113 (95.76%) had APGAR score more than six at five minutes. Out of the total live births, it was found that 107 (94.69%) live births had an APGAR score of more than six and 6 (5.30%) had scored less than six at one minute.

## DISCUSSION

Out of 640 obstetric patients who were admitted during the study period, 118 (18.4%) patients underwent induction of labour for various indications compared to 10.5% according to study done in Mid-Western Regional Hospital in Surkhet, Nepal.^4^ The sample size was proportionately larger of 3,694 compared to our study. Similar prevalence was seen in KMCTH, Kathmandu 19.70%, and Southwest Ethiopia 20.4%.^5,6^ However the prevalence was 9.96% (8.37-11.55, 95% Confidence Interval) in Birat Medical Teaching Hospital.^7^ The high volume deliveries in Terai region compared to Hilly region might attribute to the discrepancy in the finding.

In this study, the most common indication for induction of labour was post-dated pregnancy 109 (92.37%). Post-dated pregnancy was the leading indication similar other studies although accounting for lesser proportions 64.7% and 51.28%.^5,8^ However, in a study the commonest cause of indication for induction of labour was oligohydramnios (30.9%).^9^

In this study, of patients who received per oral misoprostol 48 (71.64%) had successful vaginal delivery and 19 (28.36%) had caesarean section. In contrast, among those who received sublingual misoprostol 46 (90.19%) had vaginal delivery and 5 (9.81%) had caesarean section. However in a study, women grouped for sublingual misoprostol had vaginal delivery rate of 91.25% while those for oral misoprostol had vaginal delivery rate of 92.5%.^10^ This study shows 94 (79.66%) of the patients undergoing induction of labour with misoprostol had successful vaginal delivery while 24 (20.34%) had caesarean section. Majority of the caesarean section were attributed to failed induction of labour 16 (66.67%). Similar outcome 80.5% vaginal delivery and 19.5% caesarean section was seen in a study done at Government Medical College, Aurangabad.^11^

In this study 34 (36.17%) of vaginal delivery were achieved after receiving 2 doses of misoprostol administered 6 hours apart. However according to Levy et.al., 44 (68.7%) of successful induction was achieved after 1 capsule of per oral misoprostol.^12^ According to this study among the primigravida who received misoprostol for induction, vaginal delivery was observed in 71.43% and caesarean section in 28.57%. Likewise, among multigravida 89.09% had vaginal delivery whereas 10.91% had caesarean section. Similar prevalence 32.3% of caesarean delivery was seen in primigravida in a study done at NMCTH, Biratnagar.^13^

There was no neonatal mortality observed in our study. Similar finding of 99.7% of alive outcome was seen in a study done by Lamichhane S. ^13^ According to a meta-analysis, subjects who underwent labour induction had lower perinatal mortality rate compared to expectantly managed patients (0.09% vs 0.33%) (OR 0.41; 95% Confidence Interval 0.14, 1.18) though the result was not statistically significant.^14^ Most of the babies delivered were of birthweight 2500 grams to 4000 grams (89.8%). Most common birth weight group in other studies were 2.5 kg-3.5 kg i.e., 88.76% and 3 kg-3.5 kg i.e 54.6%.^4,13^ The mean birth weight for electively induced infants was 3594+/-509 grams.^15^ This comparable birth weight can be attributed to the near-term induction of labour for post-dated pregnancy.

In the present study, 107 (94.69%) live births had APGAR scores of more than or equal to 6 at one minute and 6 (5.31%) had scores less than 6. Similar results were seen by Dhakal S i.e., 87.9% had APGAR score at 1 minute of more than or equal to 6 and 93.7% had APGAR score of more than or equal to 6 at 5 minutes.^4^ According to Dhan Bahadur Shrestha 14.5% of neonates among who had induction of labour were associated with low APGAR score at 5 minutes.^8^

This study was carried out in resource limited rural settings of Nepal where neonatal and maternal ICU setup are unavailable so most of the high-risk cases like hypertensive disorders of pregnancy, maternal heart disease, PPROM, GDM are identified earlier and referred to tertiary care centres. So, this study shows better maternal and neonatal outcomes when compared to other similar studies done in tertiary care centres.

## CONCLUSIONS

The prevalence of induction of labour was comparable with previous studies. Neonatal outcome, rate of vaginal and LSCS deliveries after induction of labour using misoprostol is comparable with other studies.
